# Malaria prevalence, prevention and treatment seeking practices among nomadic pastoralists in northern Senegal

**DOI:** 10.1186/s12936-017-2055-x

**Published:** 2017-10-13

**Authors:** Mame Cheikh Seck, Julie Thwing, Fatou Ba Fall, Jules Francois Gomis, Awa Deme, Yaye Die Ndiaye, Rachel Daniels, Sarah K. Volkman, Medoune Ndiop, Mady Ba, Daouda Ndiaye

**Affiliations:** 1 Department of Parasitology, Faculty of Medicine and Pharmacy, Cheikh Anta Diop University, Dakar, Senegal; 2 Malaria Branch, Division of Parasitic Diseases and Malaria, U.S. Centers for Disease Control and Prevention, President’s Malaria Initiative, Atlanta, GA USA; 3 Senegal National Malaria Control Program, Dakar, Senegal; 4 Harvard T.H Chan School of Public Health, Boston, MA USA

**Keywords:** Malaria, Burden, Nomadic pastoralist, Fulani, Senegal, Insecticide treated net, Access to care, Care seeking

## Abstract

**Background:**

Malaria transmission in Senegal is highly stratified, from low in the dry north to moderately high in the moist south. In northern Senegal, along the Senegal River Valley and in the Ferlo semi-desert region, annual incidence is less than five cases per 1000 inhabitants. Many nomadic pastoralists have permanent dwellings in the Ferlo Desert and Senegal River Valley, but spend dry season in the south with their herds, returning north when the rains start, leading to a concern that this population could contribute to ongoing transmission in the north.

**Methods:**

A modified snowball sampling survey was conducted at six sites in northern Senegal to determine the malaria prevention and treatment seeking practices and parasite prevalence among nomadic pastoralists in the Senegal River Valley and the Ferlo Desert. Nomadic pastoralists aged 6 months and older were surveyed during September and October 2014, and data regarding demographics, access to care and preventive measures were collected. Parasite infection was detected using rapid diagnostic tests (RDTs), microscopy (thin and thick smears) and polymerase chain reaction (PCR). Molecular barcodes were determined by high resolution melting (HRM).

**Results:**

Of 1800 participants, 61% were male. Sixty-four percent had at least one bed net in the household, and 53% reported using a net the night before. Only 29% had received a net from a mass distribution campaign. Of the 8% (142) who reported having had fever in the last month, 55% sought care, 20% of whom received a diagnostic test, one-third of which (n = 5) were reported to be positive. Parasite prevalence was 0.44% by thick smear and 0.50% by PCR. None of the molecular barcodes identified among the nomadic pastoralists had been previously identified in Senegal.

**Conclusions:**

While access to and utilization of malaria control interventions among nomadic pastoralists was lower than the general population, parasite prevalence was lower than expected and sheds doubt on the perception that they are a source of ongoing transmission in the north. The National Malaria Control Program is making efforts to improve access to malaria prevention and case management for nomadic populations.

**Electronic supplementary material:**

The online version of this article (doi:10.1186/s12936-017-2055-x) contains supplementary material, which is available to authorized users.

## Background

Senegal is a malaria-endemic country in the Sahel zone of West Africa. Malaria control interventions have been scaled up dramatically in the last decade, resulting in a substantial decrease in parasite prevalence, and possibly contributing to a significant drop in all cause under five mortality [1]. Malaria transmission in Senegal is highly seasonal, concentrated during and just after rainy season (generally July–October), peaking in October and November. Malaria incidence is moderate to high in the southeast, where it is relatively wet, decreasing toward the north. In northern Senegal, along the Senegal River, which makes up the northern border with Mauritania, and in the Ferlo Desert, annual incidence in 2016 was less than five cases per 1000 inhabitants, and in some sites under 1/1000 [2]. Even with this low incidence, a large proportion of the cases detected in the far north are among travelers from other regions. In some areas, health providers report that the majority of the cases diagnosed are among non-residents of the district [3], and in a study of reactive active case detection around index cases in one northern district, 80% of index cases had traveled in the past 15 days [4].

While reasons for such travel include business, school, and visiting family and/or friends, a group of travelers of concern to the National Malaria Control Program (NMCP) were nomadic pastoralists. Senegal, as with many countries in the Sahel, hosts a large number of nomadic pastoralists, primarily of the Fulani ethnic group, who move with their flocks to find pasture. In Senegal, these pastoralists may have more permanent dwellings in the north, but spend dry seasons in the comparatively moist south, returning north during rainy season. A large number of pastoralists move with their livestock from southern high malaria transmission zones toward the Ferlo and the Senegal River Valley every rainy season [5]. Anecdotal reports from health providers in the north suggest that the first malaria cases of the season are typically found among nomadic pastoralists, followed 2 to 3 weeks later by cases among residents. Due to their mobility, these nomads are at risk of not being included in campaign-based malaria prevention strategies, such as mass distribution of long lasting insecticide treated nets [6]. They also frequently travel in remote areas, and may have infrequent contact with the health system, sometimes only when an illness has progressed to an advanced or serious condition. No data are available regarding the burden of malaria among nomadic pastoralists of Senegal, nor are their numbers known with any degree of certainty.

The Senegal National Malaria Control Program has set an ambitious target to reach pre-elimination status by 2018 [7]. Interventions supporting malaria elimination, such as case investigation and reactive active case detection, in which all passively detected cases are investigated and household members tested for malaria, have been introduced in the northern regions of St. Louis, Louga, and Matam, covering the northern third of the country. Given the population movement of nomadic pastoralists between zones of high and low transmission, there was concern that these populations may play a role in maintaining transmission in the north, undermining malaria elimination efforts. Of particular concern was the reservoir of asymptomatic malaria infections, for which care would not be sought due to the lack of symptoms, but which could contribute to ongoing transmission. Movement of malaria parasites by human migration can quickly undermine efforts to suppress or interrupt transmission. The World Health Organization has advised endemic countries on the role that may be played by population migration on the basis of studies and assessments made in several endemic countries [8, 9]. Mobile populations may constitute a barrier to malaria elimination efforts in some regions, and may participate in the reintroduction of malaria in zones where it had been eliminated [10].

Due to their mobility, nomadic populations are very challenging to study. They are likely to be missed during census activities, and if included, are not associated with a defined geographic location where they may be reliably found. While large family groups may travel together, the population composition of any given locality where they may congregate fluctuates as family groups arrive and leave. In Senegal, when they arrive in the north during the rains, they may stay with family members who are part of the non-nomadic population. Hence, development of a probability sampling frame is extremely difficult, if not impossible. Respondent driven sampling (RDS) was developed to study hidden populations [11], and while it has historically been used to study populations such as injection drug workers and sex workers, it has been used in the Mekong to study mobile migrants in the context of malaria elimination efforts [12–14]. A variant of snowball sampling, this methodology relies on participants to recruit their contacts in the target population. It attempts to control for bias introduced by zealous recruitment by some participants compared to others by restricting the number of participants any one participant can recruit and uses the social networks described by the participants to construct a sampling frame. Analytical software has been developed to analyze datasets generated with RDS methodology. RDS requires each participant to have social networks and to recruit other participants independently. However, the need to include minors on the sample made it impossible to implement a strict RDS protocol as minors would not be able to recruit independently. Features of RDS sampling to minimize bias were retained, encouraging participants to recruit a uniform number of other participants, including at least one minor. Thus, a modified snowball sampling study was conducted in order to understand access to and use of malaria prevention interventions including health messaging and insecticide treated nets, treatment access and treatment seeking practices, and malaria parasite prevalence among nomadic pastoralists in the Senegal River Valley and in the Ferlo Desert, and to use molecular barcoding in effort to determine the origin of malaria parasites found among this population.

## Participants and methods

### Study area

**Fig. 1:**
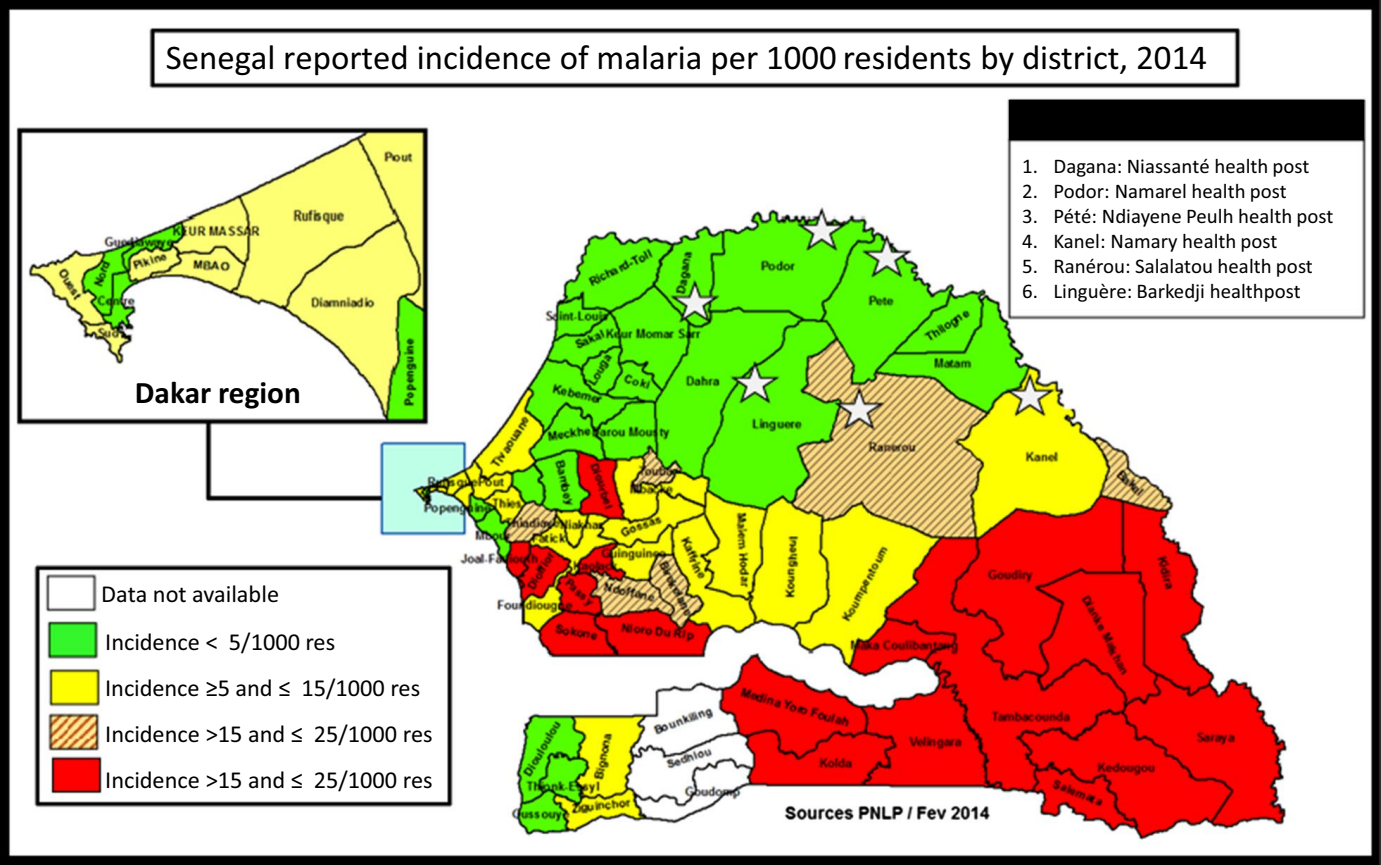
Map of Senegal with study sites overlaid on annual incidence of confirmed malaria per 1000 inhabitants by district, 2014

This study was carried out from September to October 2014 in the Ferlo Desert and the Senegal River Valley. The Ferlo Desert is a vast Sahelian plain just south of the Senegal River in northeastern Senegal of over 75,000 km^2^ with a population of 511,432 inhabitants [15, 16]. The Senegal River Valley stretches over 800 km from the coast to the border of Mali and along the Falémé tributary of the Senegal River. The population was estimated in 2013 to be 1,496,383 residents, with 75% in rural settings [16]. Study sites known to host large numbers of nomadic pastoralists during rainy season (primarily because of the presence of water sources for their livestock) were selected purposively. In each study area, three health districts were chosen: the districts of Linguere, Ranerou and Kanel in the Ferlo Desert; and Dagana, Podor and Pété in the Senegal River Valley. In the Ferlo Desert, the communities of Barkédji (Linguere), Salalatou (Ranerou) and Namary (Kanel) were selected, and in the Senegal River Valley, Niassanté (Dagana), Namarel (Podor) and Ndiayéne Peul (Pété) were selected (Fig. 1). In these study areas, the dry season is from November to June and the rainy season from July to October. The annual rainfall can reach 600 mm, increasing from north to south. Malaria transmission in these areas is highly seasonal with transmission from July to December [17]. In the Senegal River Valley, the reported annual incidence of malaria in 2014 was less than five confirmed cases per 1000 people and in the Ferlo Desert, reported annual incidence ranged from five to 25 cases per 1000, compared to over 200 cases per 1000 in the southeast [2].

### Study population

Nomadic pastoralists aged 6 months and older, who were returning or had just returned to the north from the south during rainy season were recruited (the duration of the travel between north and south may last several months). Study participants reported no acute illness, had axillary temperature < 37.5 °C, and provided written informed consent before being enrolled in the study. For children, informed consent was provided by their parents or legal guardian. Potential participants were excluded if they lived permanently in the district, were not members of the nomadic community, had an acute febrile illness, or had previously taken part in the study. Given the nature of the sampling design, with participants being recruited and coming to the interviewers to participate, it was important not to become seen as care seeking opportunity, so as not to falsely inflate the parasite prevalence among an already stigmatized population. Therefore, potential participants with acute febrile illness were excluded from the study, and offered malaria testing, with treatment if the result was positive.

### Sampling and recruitment

A snowball sampling survey, using a modified respondent driven sampling methodology was conducted, as described above. Chief nurses at the health posts that served each community and were familiar with them selected leaders in the nomadic community to serve as initial seeds that would recruit other participants. Each seed was asked to recruit three other individuals, including one participant under the age of 15 years, so as to include children in the sample. While this strategy made the sample more representative of the target population, it made it impossible to use classic RDS methodology and analysis, which depends on each participant having an independent social network from which they can recruit. We did not discourage recruitment of household members. Interviews were held at a designated central location chosen to facilitate participation. Each subsequently recruited participant received the same instructions, with recruitment continuing until the sample size of 1800 was reached (300 in each of the six sites). To calculate sample size, a target proportion of 50% for key variables such as care seeking and net use, with a confidence level of 95% and a confidence interval of (0.40, 0.60), and a design effect of 3 were used, giving 300 participants per site, a total of 1800 participants.

### Data collection

After informed consent, a questionnaire was administered for collecting socio-demographic data (age, gender, ethnicity, and literacy), knowledge of malaria prevention and treatment, ownership and use of insecticide treated nets, care seeking practices for illness within the last month, recent travel history, and plans for future travel (Additional file 1). Because data were collected at a central location rather than in the household, treatment status of the bednet was not possible to ascertain. When the participant was a child under 15 years of age, the caregiver answered the questionnaire. Axillary temperature was measured by study staff.

### Laboratory methods

#### Rapid diagnostic test, thick and thin smears

Capillary blood samples were collected by study staff using finger prick. One drop of blood was used to perform a malaria rapid diagnostic test (RDT) (SD BIOLINE Malaria Ag P.f., Standard Diagnostics, Korea) and two drops for the thin and thick smear. For molecular analysis, three to four drops of blood were spotted on Whatman 903 protein saver card (Sigma-Aldrich, St. Louis, MO, USA) filter paper and dried at room temperature for further extraction of *Plasmodium* DNA. Filter papers were sealed in plastic bags with silica gel and kept at room temperature until DNA extraction. Thin smears were fixed with methanol and all smears were stained with 10% Giemsa for 15 min. Microscopy was used to detect absence or presence of asexual and sexual parasites. Parasite density was determined by counting the number of asexual parasites per 200 to 500 white blood cells, and calculated per µl using the following formula: number of parasites × 8000/200 − 500 assuming a white blood cell count of 8000 cells per µl. An absence of malaria parasites in 250 high power ocular fields of the thick film was considered negative. Quality control was performed by having a different experienced laboratory technician read 10% of all slides; in case of discrepancies, a third independent reading was performed and the average of the results obtained by the two closest readings was recorded.

#### Molecular diagnostic testing ssr RNA gene amplification by polymerase chain reaction (PCR)

DNA was extracted from whole blood with the QIAamp DNA blood mini kit (Qiagen Inc., Valencia, CA, USA). The ssrRNA gene was amplified as previously described [18, 19] with minor modifications. First round PCR to detect *Plasmodium* genus was performed using the following primers rPLUf 5′-TTA AAA TTG TTG CAG TTA AAA CG-3′ and rPLUr 5′-CCT GTT GTT GCC TTA AAC TTC-3′. The second round PCR was performed with primers specific to four *Plasmodium* species:

**Table:** 

*Plasmodium malariae*	rMALf5′-ATA ACA TAG TTG TAC GTT AAG AAT AAC CGC-3′ rMAL5′-AAA ATT CCC ATG CAT AAA AAA TTA TAC AAA-3′
*Plasmodium falciparum*	rFALf 5′-TTA AAC TGG TTT GGG AAA ACC AAA TAT ATT-3′ rFALr 5′-ACA CAA TGA ACT CAA TCA TGA CTA CCC GTC-3′
*Plasmodium ovale*	rOVAf 5′-ATC TCT TTT GCT ATT TTT TAG TAT TGG AGA-3′ rOVAr 5′-GGA AAA GGA CAC ATT AAT TGT ATC CTA GTG-3′
*Plasmodium vivax*	rVIVf 5′-CGC TTC TAG CTT AAT CCA CAT AAC TGA TAC-3′ rVIVr 5′-ACT TCC AAG CCG AAG CAA AGA AAG TCC TTA-3′

PCR reactions were performed in a final volume of 20 μl: GoTaq (Promega Green Master Mix 2X) 6 μl, forward primer 10 pmol/μl (1 μl), reverse primer 10 pmol/μl (1 μl), DNA 2 μl, and 10 μl of distilled water. The PCR program was as follows: initial denaturation at 94 °C for 4 min, then 35 cycles at 94 °C for 30 s, 55 °C for 1 min (first round annealing) or 58 °C for 1 min (second round annealing), 72 °C for 1 min, final extension at 72 °C for 4 min, and 4 °C continuous at the end.

#### DNA genotyping and sequencing by high resolution melting

Samples were analyzed to determine if they were monogenomic and genetically distinct using a 24 single nucleotide polymorphism (SNP) molecular barcode, as described elsewhere [20, 21].

### Statistical methods

Excel (Microsoft Office 2007, Seattle, WA, USA) was used for data entry, and statistical analysis was performed using STATA version IC 12 (College Station, TX, USA) and Epi-Info 7 (CDC, Atlanta, GA, USA). Given that this was a non-probability sample (resulting from the need to include minors, and thus the inability to use RDS analytic software), proportions were calculated for categorical data, and means for continuous data, but confidence intervals were not presented.

### Ethical considerations

The project was discussed with community members, health post chief nurses, and district medical officers prior to the study, and community consent was obtained from community leaders. Written informed consent was obtained from all participants or guardians prior to their enrollment. The study protocol received a non-research determination from CDC Atlanta (Approval No. CGH HSR#:2014-193, 27 August 2014) and was approved by the ethical review committee of the Ministry of Health, Senegal (Approval No. 324/MSAS/DPRS/CNERS, 26 August 2014). Participation was strictly voluntary, and patients who were ineligible due to fever were offered diagnosis and treatment free of charge, as indicated by national policy.

## Results

### Enrollment

In the six study districts in the Senegal River Valley and the Ferlo, 1854 participants were screened and 1800 included (300 participants per district). Fifty-four were excluded from the study: 48 were ineligible (45 febrile and three permanent residents) and six refused to participate. Fever was the only cause of ineligibility in five of the six sites; in Ranerou, three permanent residents presented for screening. The six cases of refusal were attributed to fear among children (Table 1). Those ineligible to participate due to fever were offered diagnostic testing with treatment as indicated by the results.

**Table 1:** Distribution of participants according to the site

Areas	Ferlo			Senegal River Valley			Total
District	Kanel	Ranerou	Linguere	Podor	Dagana	Pété	
Health post	Namary	Salalatou	Barkedji	Namarel	Niassante	Ndiayene Peul	
Screened	313	307	306	316	304	308	1854
Ineligible	12	5	4	16	4	7	48
Refuse	1	2	2	0	0	1	6
Participants	300	300	300	300	300	300	1800

### Characteristics of the study population

The mean age of participants was 25.1 years (range 1–85 years); 61% were male. Children under 15 years constituted approximately one-third (32%) of participants, in accordance with the recruitment instructions, 13% were aged 15–20 years, and 56% were older than 20 years. The majority (88%) belonged to the Pulaar ethnic group, also known as Peul, Fula, or Fulani, a primarily pastoralist ethnic group found across the Sahel. The Serer ethnic group represented 12%, and was found exclusively in the Linguere district. The majority was illiterate; only 4% could read and understand French, and 2% could read and understand their language in Arabic script.

### Health messaging

Among nomadic pastoralists, 73% had heard of malaria. While 44% said they had received health messages (from any source) in the last 3 months, 29% had received health messages specifically related to malaria. However, of those who responded (n = 1563), 87% knew the importance of sleeping under a bednet; 38% knew that pregnant women should take sulfadoxine-pyrimethamine (SP) during pregnancy to prevent malaria; and 45% knew about the importance of seeking care for malaria in a timely manner. The most common source of messages was radio, which was a means of receiving health messages for 93% of those who reported receiving them.

### Ownership and use of bednets

Though 64% reported having at least one bednet in their household, only 29% reported having received a net from a mass distribution campaign. Overall, 38% reported having received a net at a health facility, with 52% saying they paid for it and 48% saying they received it for free. Of the 33% who had purchased a net somewhere other than a health facility, 81% purchased it at a weekly market. Of those who responded to a question regarding the most convenient place to obtain a bednet (n = 1676), 45% said a health facility, followed by 25% at a weekly market, while 14% wanted the opportunity to purchase a net from home. Of all participants, 53% reported having used a bednet the night before the survey, 26% reported using them every night, and 61% used one some nights. The primary reason for not using a bednet every night (40%) was lack of access to one, followed by lack of mosquitoes (39%).

### Care seeking and treatment

Among the 1792 who responded, 8% (n = 142) reported having suffered a febrile illness in the previous month. Of these, 27% reported taking no action, while 55% sought care either at a formal health facility or from a community health worker (32% at a health facility and 29% at the community level, with some going to both). While 23% had self-medicated at home (a third of whom also sought care), only 6% sought the services of a traditional practitioner (half of whom also sought care elsewhere). Of those that did not seek care and answered the question as to why they did not (n = 56), 36% said it was too far, 25% lacked sufficient funds, 16% said it was unnecessary, and 16% did not have time. Of all those who sought care, 20% reported that a malaria diagnostic test had been done. Of those who knew a diagnostic test had been performed (n = 17), five reported that the result was positive, three of whom reported being treated with an ACT (two of whom also reported receiving an injection or intravenous medication), and two of whom were unsure what they received.

While 404 (23%) reported having suffered an illness of any kind in the last month, 329 responded as to what they did for that illness: 42% went to a formal health facility, 7% went to a community level provider, 7% went to a pharmacy or informal drug vendor, 26% sought no treatment, and 13% took medicines they had at home. The primary means of transportation for care seeking was a horse or donkey cart (75%), followed by walking (15%), and while the trip took an hour or less for 48%, it took two or more hours for 21%. When asked about challenges in seeking care, 67% stated that it was too far. While there were few complaints as to the treatment received once there, 95% stated that they would favor training a member of their group to perform case management using RDT and ACT.

### Migration patterns

Most participants were recently arrived, with 15% having arrived in the last 2 weeks, 55% having arrived within the last 4 weeks, 76% within the last 8 weeks, and 96% within the last 12 weeks. Planned duration in the district was short, with 61% planning to stay 3 months or less, and 93% planning to stay 4 months or less. Only 38% said they followed the same migration patterns every year. While participants were asked from where they had come and to where they were going, the inability to the connect the location names given with known place names made analyzing this information impossible.

### Prevalence of malaria infection among nomadic pastoralists

Among the 1800 blood samples tested for malaria, 11 were positive by RDT, eight by thick smear, and nine by PCR; *Plasmodium falciparum* was the only malaria species detected. All that were positive by thick smear were also positive by RDT and PCR, and all that were positive by PCR were also positive by RDT. There were two RDT positives that were negative by PCR and thick smear. Malaria positive individuals (by PCR) were exclusively found in the districts of Kanel (n = 3), Ranerou (n = 3), and Pété (n = 3) (Table 2). One had come from Mali, but the others had come from within Senegal, and eight of the nine had arrived four or more weeks earlier. Parasite density ranged from 200 to 2000/μl. All positives were among members of the Pulaar ethnic group.

**Table 2:** Annual reported incidence by district from the routine malaria information system and detected prevalence of malaria among nomadic pastoralists

District	Annual reported incidence in 2014 per 1000 inhabitants^a^	Number examined	Number infected by		
			RDT n (%)	Thick smear n (%)	PCR n (%)
Ferlo					
Kanel	6.7	300	4 (1.3)	2 (0.7)	3 (1.0)
Ranerou	20.0	300	3 (1)	3 (1)	3 (1)
Linguere	3.4	300	0 (0)	0 (0)	0 (0)
Senegal River Valley					
Podor	1.0	300	0 (0)	0 (0)	0 (0)
Dagana	0.4	300	0 (0)	0 (0)	0 (0)
Pété	1.9	300	4 (1.3)	3 (1.0)	3(1.0)
Total	4.0	1800	11 (0.6)	8 (0.4)	9 (0.5)

^a^Data reported by Senegal National Malaria Control Program, including all publicly supported health facilities in each district

Of those who were ineligible to participate due to febrile illness, 7/45 (16%) were positive by RDT: one in Pété (1/7), four in Kanel (4/12), and two in Ranerou (2/2), the same districts in which there were asymptomatic positives. In the Linguere, Dagana, and Podor sites, none of the 24 febrile potential participants had positive RDTs. If these patients are included in the overall total, parasite prevalence by RDT would be 0.98%; blood slide and PCR results are not available as these individuals did not undergo informed consent, but were offered case management for fever.

### Molecular barcodes

Seven *P. falciparum* isolates gave analyzable barcodes (Table 3). None were clonal. These barcodes were compared to a database of over 1500 *P. falciparum* parasites isolated from different regions of Senegal: Thies, Kedougou, Kolda, Dakar, Kaolack, Kaffrine, Saint Louis and Velingara. Except for one isolate (D076), which showed an incomplete partial similarity (22/24) to an isolate from Thies in 2012 (Table 4), none of the molecular barcodes identified among the migrants had been previously identified in Senegal.

**Table 3:**
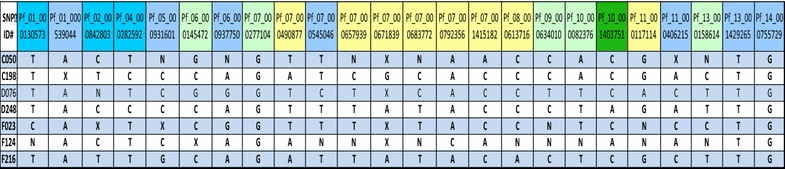
Molecular barcodes of *Plasmodium* isolates

**Table 4:**
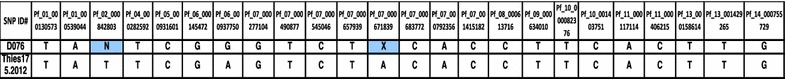
Comparison of two molecular barcodes D076 and Th175

## Discussion

This is the first study in Senegal addressing malaria among nomadic pastoralists, a group about whom little is known and who are often stigmatized. As anticipated, access to health messaging, malaria prevention (primarily bednets), and prompt and effective case management was low. Contrary to expectations, the prevalence of malaria was very low. None of the barcodes had been previously identified among a library of over 1500, giving no information as to parasite origin.

Senegal conducts a continuous Demographic and Health Survey (cDHS), with annual results and a period of data collection covering January to October, largely during low transmission season. For the year of this survey (2014), the cDHS reported that 74% of households owned at least one ITN, 46% slept under a bednet the previous night (40% under an ITN), and treatment was sought for 54% of children under 5 years who had fever in the previous 2 weeks [22]. We found ownership of any bednet to be 64%. Many of these were likely untreated given that less than half said they had received a net at a health facility, and the majority of those who said they had purchased a net elsewhere had purchased a net at a weekly market. Net use the previous night in this study was higher than that reported in the cDHS, though the study was conducted during rainy season, when use is at its highest. Care seeking for febrile individuals of any age was 55%, which was higher than had been anticipated for this population. Challenges with the distance to travel to seek care were mentioned by the majority of the nomadic population, with most seeking care either by horse or donkey cart or on foot, and more than half requiring more than an hour to reach care. While the proportion who received a diagnostic test was low, this may be in part explained by the policy in place at the time of not testing febrile patients with evidence of another fever source (cold symptoms, rash, etc.) in a context of low malaria incidence. The NMCP has since instituted a policy of universal testing of febrile patients. The number of those who reported a positive test was too small to draw conclusions regarding appropriate treatment. Health problems and lack of access to health care among nomadic pastoralists and among mobile populations in general have been frequently studied [23]. Infant and child mortality have generally been found to be higher among nomadic than settled peoples [24–26]. Insecticide treated net access has been noted to be low [27], as well as vaccination coverage [28–30].

The cDHS 2014 found parasite prevalence among children under 5 years in northern Senegal was 0.1%, compared to 5.9% in southern Senegal, the region in which nomadic pastoralists spend the dry season [22]. Given the time period of data collection for cDHS (January to October), and peak malaria transmission in October and November, with very high parasite prevalence often seen through December, the majority of cDHS samples were collected when parasite prevalence would be expected to be lower than this study, which was conducted from September 10 to October 10. This study found an overall parasite prevalence of 0.5% by PCR. Even if the individuals excluded from enrollment due to fever (n = 45) are included, with the seven positives added to the total, parasite prevalence remains below 1%. The Senegal NMCP reported annual confirmed malaria incidence per 1000 of 20.0, 6.7, and 1.9 for Ranerou, Kanel, and Pété, respectively, the three districts in which we found parasites among the nomadic population, while they reported annual confirmed malaria incidence per 1000 of 3.4, 1.0, and 0.4 in Linguere, Podor, and Dagana, the districts in which we did not find parasites [2]. In general, we found parasites among nomadic pastoralists in districts in which reported incidence among the overall population was higher. While it is not possible to conclude with certainty that the parasite prevalence among nomadic pastoralists is the same as that among residents of northern Senegal, it was surprisingly low. These findings, together with the lack of any barcodes matching those previously observed in southern Senegal, do not provide evidence to support the concern that nomadic pastoralists constitute a major source of malaria transmission in the north upon their arrival. They may leave their dry season forage before transmission season starts and thus face little malaria exposure during their sojourn in the south. Given previous reports, despite their anecdotal nature, further investigation is needed, possibly at the health facility level to study confirmed cases.

While in this sample, participants with malaria infection were only found among Pulaar (Fulani), lower susceptibility to malaria among Fulani than among sympatric ethnic groups was noted first in 1995 in Burkina Faso [31] and subsequently in Mali [32] and Sudan [33]. Fulani have been shown to have higher antibody prevalence and levels (both IgM and IgG) than sympatric populations that have similar malaria exposure [34–36]. Fulani have also been observed to have a high proportion of type-O blood, elevated pro-inflammatory cytokines, and protective IL4, Tcell, and FcγR gene polymorphisms [32, 37–43].

The study team noted that the nomadic pastoralists among whom the study was conducted were exceptionally welcoming, understanding of the importance of malaria control, and open to collaboration; the primary difficulties in conducting the study were the mobility of the population and very difficult access by vehicle, particularly after rains. This group is often stigmatized and socially marginalized, an attitude which may hamper attempts at outreach and malaria control [44]. Constructive attempts at engagement and problem solving with communities are likely to be far more effective. A community-directed intervention strategy implemented in Nigeria among Fulani pastoralists improved appropriate malaria management in children under 5 years from 2.7 to 82% [45]. Given the expressed desire of the great majority of participants to have a member of their community trained as a community health worker to diagnose and treat malaria with RDTs and ACTs, the Senegal NMCP is exploring ways to implement this strategy. In 2016, the NMCP conducted a universal coverage long lasting insecticidal net distribution, and included special efforts to reach the nomadic population. Health post nurses are requested to implement outreach visits on a regular basis to target nomadic populations. In addition to these approaches, the accessibility of radio indicates that this is an important means with which to reach nomadic pastoralists with health messages.

There are number of possible limitations in a study of this nature. While we tried to compensate for some of the innate limitations of snowball sampling by limiting the number of participants one person could recruit, the sample is not a probability sample and may not be representative. Some information collected routinely by household surveys (bednet treatment status) was not possible to obtain. We did not collect information on household composition, and were unable to determine if two respondents were members of the same household. We were also not able to use the information given to map travel routes, and thus were unable to learn about risk for origin and destination points. We were also unable to directly compare parasite prevalence with that of sympatric populations, either in the north or the south. The study was not designed to investigate symptomatic cases of malaria at the health posts; this may be of interest as a follow up study. We did not recruit and enroll febrile patients, not wanting the study to be seen as an opportunity to seek care, and thus leading to enrollment of a large number of febrile participants. Due to this decision, we did not collect filter paper on and were not able to perform molecular analysis for the seven individuals who were ineligible due to acute febrile illness and who had a positive RDT. While none of the barcodes matched others collected in higher transmission zones of Senegal, these areas have not been comprehensively mapped, and it may be possible, as further molecular studies are completed, to understand the genomic background more comprehensively.

While malaria prevalence among this population appears to be low, this may also indicate low levels of exposure. Serologic studies have not been done on this population to determine antibody responses and levels, and while susceptibility to uncomplicated malaria may be reduced among the Fulani, relative risk of severe malaria has not been studied, and malaria epidemics have been documented in other nomadic pastoralist groups [46]. Efforts to reach this population with health messages, insecticide treated nets, and prompt effective case management are critical, both to prevent morbidity and mortality among this population, and to reach malaria elimination.

## Conclusions

This study examined malaria prevention, care and treatment seeking behavior, and parasitemia among asymptomatic nomadic pastoralists in northern Senegal. Low access to health messaging and insecticide treated nets, and challenges with care seeking underlines the need to design strategies to reach this group as Senegal moves toward elimination. The low prevalence of malaria infection among this group suggests that they may be at risk of severe disease. Further investigation is needed to clarify to what extent they may contribute to ongoing transmission in northern Senegal, and to understand the burden of symptomatic malaria among this population.

## Additional file

**Additional file 1.** Participant questionnaire.
