# Supplementary material for: Malaria prevalence, prevention and treatment seeking practices among nomadic pastoralists in northern Senegal
Source: Malar J. 2017 Oct 13;16:413. doi: 10.1186/s12936-017-2055-x (PMC5640937; doi:10.1186/s12936-017-2055-x)
Supplement: Supplementary file 1 — Additional file 1. Participant questionnaire. [file 12936_2017_2055_MOESM1_ESM.docx]

**Questionnaire**

1. Health district ___________________
2. Health post ____________________
3. Date___________________________
4. Age _________ yrs
5. Gender : M F
6. Ethnic group:

A) Pulaar

B) Other, specify _______________

1. Telephone number if any _______________________
2. Education received:

A) French school primary

B) French school secondary

C) Koranic school

D) None

1. Can the person read and understand French? (sample text provided) (Y/N)
2. Can the person read and understand Pulaar in Arabic script? (sample text provided) (Y/N)

**Malaria prevention**

1. Do you know what malaria is?
   1. Yes
   2. No
   3. Don’t know
2. Do you think you and your family are at risk of malaria?
   1. Yes
   2. No
   3. Don’t know
3. Do you think it is possible to prevent malaria?
   1. Yes
   2. No
   3. Don’t know
4. Do you know methods to prevent malaria?
   1. Insecticide treated nets
   2. Indoor residual spraying
   3. Mosquito coils
   4. Mosquito spray (Yotox)
   5. Taking medicine to prevent malaria regularly
   6. Herbal tea
   7. Other, specify ____________________
5. What, if any, methods do you use to prevent malaria?
   1. Insecticide treated nets
   2. Indoor residual spraying
   3. Mosquito coils
   4. Mosquito spray (Yotox)
   5. Taking medicine to prevent malaria regularly
   6. Herbal tea
   7. Other, specify ____________________
6. Do you have any insecticide treated nets in your household?
   1. Yes
   2. No
   3. Don’t know
7. Have you ever received any Insecticide treated nets free of charge during mass distribution campaign? (if #17 yes, then #18; else #20)
   1. Yes
   2. No
   3. Don’t know
8. If so, in which district? _______________
9. In what year? (2008, 2009, 2010, 2011, 2012, 2013, 2014)
10. Have you ever received an insecticide treated net from a health facility? (if #20 = yes then 21, else 22)
    1. Yes
    2. No
    3. Don’t know
11. Did you pay for it or was it free?
    1. Paid
    2. Free
    3. Don’t know)
12. Have you ever purchased an insecticide treated net somewhere other than a health facility? (if yes then #23, else 24)
    1. Yes
    2. No
    3. Don’t know
13. Where?
    1. Pharmacy
    2. Weekly market
    3. Shop
    4. Other, specify ____________
14. If you have never purchased an insecticide treated net, why?
    1. I don’t need one
    2. I don’t have the money
    3. I don’t know where to buy one
    4. I use other methods to prevent malaria
    5. Other, specify _________________
15. Where would you like to see insecticide treated net available for purchase?
    1. Pharmacy
    2. Weekly market
    3. Shop
    4. Other, specify ____________
16. Did you sleep under an insecticide treated net last night?
    1. Yes
    2. No
17. Do you sleep under a net: (if not A, then #28, else #29)
    1. Every night
    2. Some nights
    3. Never
18. Why do you not sleep under a net every night?
    1. I don’t have a net
    2. Too hot
    3. No mosquitoes
    4. Inconvenient
    5. Challenging to hang net
    6. I don’t like sleeping under a net

**Care seeking (we will ask first about your family, then about you personally)**

1. Has anyone in your immediate family been sick with a fever in the last month? (if more than one, select the one most recently ill) *(if no , go to Q44)*
   1. Yes
   2. No
   3. Don’t know
2. If so, how is the sick person related to you?
   1. Self
   2. Spouse
   3. Father
   4. Mother
   5. Sister
   6. Brother
   7. Child
   8. Other
3. Concerning the sick person, did they take medicine at home or from a neighbor?
   1. Neither
   2. Home
   3. Neighbor
   4. Don’t know
4. Did the sick person consult a traditional practitioner?
   1. Yes
   2. No
   3. Don’t know
5. Did the sick person go to a health hut or DSDOM?
   1. Yes, Health hut
   2. Yes, DSDOM
   3. Yes, A community health agent, but don’t know what kind
   4. No - neither
   5. Don’t know

1. Did the sick person go to a health post? (if Q33 or Q34 = yes, then Q35, else Q36)
   1. Yes
   2. No
   3. Don’t know
2. How long did the sick person have symptoms before they were taken to a health hut, DSDOM, or health post? (skip to Q39)

__________hours OR __________days

1. If you did not take the sick person to a health hut, DSDOM, or health post, why?
   1. No money
   2. It was too far
   3. It was not necessary
   4. We did not have the time
   5. We didn’t know where to go
   6. Other, specify _______________
2. If they sought care, but not with any of these, where did they seek care?

Text ____________________

1. Did the sick person receive a test for malaria? (If Q38 = yes, then Q39, else Q41)
   1. Yes
   2. No
   3. Don’t know
2. Was the test for free or after payment?
   1. Free
   2. Paid
   3. Don’t know
3. What was the result of the test for malaria?
   1. Positive
   2. Negative
   3. Don’t know
4. Did the person receive medicine for malaria? (if yes then Q42, else q44)
   1. Yes
   2. No
   3. Don’t know
5. Where did the person receive the medicine?
   1. Health post
   2. Health hut / ASC
   3. DSDOM
   4. Private pharmacy
   5. Small shop
   6. Other vendor (vendeur ambulant)
   7. Other specify ______________
6. What type of medicine did the person receive? (check all that apply)
   1. Antimalarial – ACT
   2. Antimalarial - quinine
   3. Antimalarial – other
   4. Antimalarial – don’t know
   5. Antibiotic
   6. Antipyretic
   7. Vitamin/iron
   8. Other, specify __________________
   9. Don’t know
7. What are the difficulties in seeking care?
   1. It’s far to go
   2. It’s expensive
   3. I don’t know where to go
   4. The people at the health posts don’t speak my language
   5. Other, specify ______________
8. Have you had any illness in the last month? (if no or don’t know, skip to Q56)
   1. Yes
   2. No
   3. Don’t know
9. Where did you go for treatment?
   1. Took medicine at home
   2. Took medicine from a neighbor
   3. Consulted a traditional practitioner
   4. Went to a health hut
   5. Went to a DSDOM
   6. Went to a health post or health center
   7. Went to a pharmacy
   8. Went to other vendor (vendeur ambulant)
   9. Other, specify _______________
10. When was the last time you consulted at a health post? (month/year)
11. How long did it take you to get there?
    1. Less than 30 minutes
    2. At least 30 minutes but less than 1 hour
    3. At least one hour but less than two hours
    4. At least 2 hours but less than 3 hours
    5. More than 3 hours
12. How did you travel there?
    1. On foot
    2. Donkey or horse cart
    3. Donkey or horse back
    4. Bicycle
    5. Motorcycle
    6. Car or truck
13. Were you able to easily find where you needed to go?
    1. Yes
    2. No
14. Were you able to understand the health worker and were they able to understand you?
    1. Yes
    2. No
15. Did they treat you politely?
    1. Yes
    2. No
16. Did they explain the treatment to you?
    1. Yes
    2. No
17. Would you return to that health post if you are sick again and in the same place?
    1. Yes
    2. No
18. If not, why? (reasons? Or text?)
19. Where would you prefer to go for treatment if you were ill?
    1. Health post
    2. Health hut / ASC
    3. DSDOM
    4. Private pharmacy
    5. Small shop
    6. Other vendor (vendeur ambulant)
    7. Other specify ______________
20. If a member of your community is trained to diagnose malaria with a blood test and give treatment, would you consult that person?
    1. Yes
    2. No

**Health messages**

1. Have you received any health messages in the last three months? (if no, skip to Q64)
   1. Yes
   2. No
2. What topics did you receive messages on? (check all that apply)
   1. malaria
   2. nutrition
   3. pneumonia
   4. diarrhea
   5. TB
   6. HIV
   7. malnutrition
   8. prenatal care/pregnancy
   9. family planning
   10. vaccination
   11. other
3. How did you receive the messages?
   1. health worker
   2. community leader
   3. neighbor/family member
   4. religious leader
   5. radio
   6. poster
   7. other, specify _______________
4. Did you receive any messages on malaria prevention or treatment? (if no skip to Q64)
   1. Yes
   2. No
5. What malaria topics?
   1. How malaria is transmitted/what causes malaria
   2. Importance of a bednet in preventing malaria
   3. Hanging your bednet
   4. Sleeping under your bednet every night, all year
   5. Every member of the family should sleep under a bednet
   6. How to care for your bednet
   7. Importance of seeking care early if you have a fever
   8. Pregnant women should take SP to prevent malaria
6. How did you receive the messages?
   1. health worker
   2. community leader
   3. neighbor/family member
   4. religious leader
   5. radio
   6. poster
   7. other, specify _______________

**Movements**

1. How long have you been in this district?

_________days or _________weeks

1. How long do you plan to stay?

_________days or _________weeks

1. Where were you before you came to this district?
   1. district: ______________________
   2. if not Senegal, what country: __________________
2. Where do you plan to go when you leave here?
   1. district: ______________________
   2. if not Senegal, what country: __________________
3. Where do you spend dry season?
   1. district: ______________________
   2. if not Senegal, what country: __________________
4. Where do you spend rainy season?
   1. district: ______________________
   2. if not Senegal, what country: __________________
5. Do you pass the same way every year?
   1. Yes
   2. No
